# Sustainable and integrated cascade valorization of the biomass generated by the invasive plant *Flaveria bidentis*: optimization of ultrasound-assisted phenols extraction coupled to the green synthesis of biogenic antimicrobial silver nanoparticles

**DOI:** 10.3389/fbioe.2026.1872480

**Published:** 2026-08-19

**Authors:** Paulina Falletti, María F. Barrera Vázquez, Elia S. Mendoza Ocampo, Paulina L. Páez, Raquel E. Martini, Patricia Lema, Laura R. Comini

**Affiliations:** 1 Facultad de Ciencias Exactas Físicas y Naturales, Universidad Nacional de Córdoba, Córdoba, Argentina; 2 Instituto de Investigación y Desarrollo en Ingeniería de Procesos y Química Aplicada (IPQA-CONICET), Córdoba, Argentina; 3 Unidad de Investigaciones y Desarrollo en Tecnología Farmacéutica (UNITEFA - CONICET), Facultad de Ciencias Químicas, Universidad Nacional de Córdoba, Córdoba, Argentina; 4 Instituto de Ingeniería Química, Facultad de Ingeniería, Universidad de la República (UDELAR), Montevideo, Uruguay; 5 Consejo Nacional de Investigaciones Científicas y Técnicas (CONICET), Córdoba, Argentina; 6 Centro de Excelencia en Productos y Procesos Córdoba (CEPROCOR), Córdoba, Argentina

**Keywords:** antimicrobial activity, antioxidant compounds, biogenic silver nanoparticles, green ultrasound-assisted extraction, optimization

## Abstract

**Introduction:**

This study addresses the integrated valorization of residual biomass from Flaveria bidentis, an invasive species native to the Americas through the recovery of phenolic compounds (phenolic acids and flavonoids, mainly glycosylated and sulfated – the latter being rare in nature –) and their subsequent use in the green synthesis of silver nanoparticles. The optimization of ultrasound‐assisted aqueous extraction of phenolic compounds from F. bidentis biomass and the direct use of the resulting extracts as reducing and stabilizing agents for the green synthesis of silver nanoparticles remain largely unexplored.

**Methods:**

The phenolic compounds were extracted by ultrasound‐assisted extraction (UAE) using water as extraction solvent. Extraction time, temperature, and solvent/sample ratio were optimized within the investigated experimental domain using response surface methodology based on a three‐variable Doehlert experimental design (R2 ≥ 0.96). The optimized aqueous extracts were then used directly, without purification, as reducing and stabilizing agents in the green synthesis of silver nanoparticles. These nanoparticles were characterized in terms of size and morphology, and their antibacterial activity against Gram‐ positive and Gram‐negative bacteria was subsequently evaluated using the diffusion assay.

**Results and Discussion:**

Optimal conditions for phenols extraction were established from leaves (68 min, 59 °C, 20 mL/ g) and from flowers (65 min, 75 °C, 20 mL/g). Under these conditions, the results revealed a key finding: the high yield of phenolic compounds from F. bidentis, particularly from the leaves (250.86 mg/g), using water as the sole solvent. Spherical biogenic silver nanoparticles (∼30–31 nm; PDI: 0.26–0.46; ζ‐potential: −15.0 to −12.1 mV) were successfully synthesized and exhibited significant antimicrobial activity. The results demonstrate the feasibility of integrating phenolic extraction and nanoparticle synthesis into a single process, in which the aqueous extracts obtained under optimized conditions can be directly employed to produce functional nanomaterials without intermediate purification steps. Thus, the proposed cascade valorization strategy enabled the sustainable transformation of *F. bidentis* biomass into bioactive compounds and antimicrobial nanomaterials, adding value to this plant waste.

## 1 Introduction

*Flaveria bidentis* (L.) Kuntze (Asteraceae), commonly known as fique, contrayerba, chasca, balda, or matagusanos, is an annual invasive weed native to South America, mainly Argentina, that has become widely distributed worldwide (Xie et al., 2010).

This plant is considered invasive due to its high reproductive and survival capacity, which negatively affect native ecosystems (Xie et al., 2010; Zhang et al., 2017). In particular, it poses a serious threat to crop growth, causing significant economic losses, in addition to generating large volumes of biomass as a result of weeding (Sanchez, 2010; Xie et al., 2010; Zhang et al., 2017). From an agro-industrial perspective, this species has a negative impact on farmers; therefore, its valorization could provide an opportunity to transform this problematic biomass into value-added products.

Several studies have shown that many of its active metabolites belong to family of phenolic compounds (mainly flavonoids and phenolic acids), a group of highly relevant and abundant secondary metabolites (Teles et al., 2018; Dzah et al., 2020). The chemical profile of this plant has been previously studied by our research group, revealing the presence of quercetin derivatives with one of the highest degrees of sulfation reported: 3,7,3′,4′-quercetin tetrasulfate and Quercetin 3-acetyl-7,3′,4′-trisulfate (rare in nature) (Pereyra de Santiago and Juliani, 1972; Cabrera and Juliani, 1976; Cabrera and Juliani, 1977; Cabrera and Juliani, 1979). This plant also contains other mono- and di-sulfated derivatives of quercetin and isorhamnetin, as well as aglycone flavonoids and their glycosylated and methylated derivatives (Cabrera and Juliani, 1976; Cabrera and Juliani, 1977; Cabrera and Juliani, 1979; Suárez et al., 1979; Varin et al., 1986; Xie et al., 2010; Wei et al., 2011; Wei et al., 2012; Xie et al., 2013; Shaheen et al., 2015). *F. bidentis* also contains kaempferol aglycone and its glycosylated derivatives, as well as the presence of phenolic acids such as chlorogenic and caffeic acids (Xie et al., 2013; Lu et al., 2013; Shaheen et al., 2015; Páez et al., 2019). However, these compounds have generally been extracted using conventional techniques, such as reflux or soxhlet extraction, without optimization of the extraction conditions. In recent years, growing attention has been directed toward green extraction technologies aimed at enhancing the recovery of natural products while reducing energy consumption, processing time, and the use of organic solvents. Among these technologies, ultrasound-assisted extraction (UAE) has emerged as a promising and sustainable alternative (Decker et al., 2026). UAE relies on the propagation of ultrasonic waves that generate alternating high- and low-pressure cycles within the extraction medium. These cycles induce the formation, growth, and subsequent collapse of cavitation bubbles, which disrupt plant cell structures, facilitate solvent penetration into the matrix, and enhance the mass transfer of target compound (Wen et al., 2018; Antunes Filho et al., 2023). As a result, UAE can significantly improve extraction efficiency compared with conventional techniques. Furthermore, UAE is a simple and versatile technique that requires relatively low solvent volumes and short extraction times while achieving high extraction yields. These advantages make it a cost-effective, energy-efficient, and environmentally friendly approach for the recovery of bioactive compounds from plant materials (Kumar et al., 2021; Antunes Filho et al., 2023).

Phenolic compounds are well known for their antioxidant activity, a property that makes them particularly suitable for the green synthesis of metallic nanoparticles (NP). Among these noble metals nanoparticles, including silver and gold, are the most extensively studied (Zuhrotun et al., 2023) because of their great functional versatility and wide range of applications including catalysis, biological marking, drug transport, wastewater treatment, crop protection, among others (Mittal et al., 2013; Abdullah et al., 2025). In addition, metallic NP have received significant attention owing to their biological properties such as anticancer, larvicidal and antimicrobial properties (Díaz-García et al., 2019; Scolari et al., 2019). This is relevant since several bacterial pathogens have developed antibiotic resistance due to the inappropriate and excessive use of antibiotics (Chahar et al., 2018). In this context, metallic NP has emerged as a promising alternative to conventional antibiotics offering new strategies for combating bacterial pathogens (Chahar et al., 2018).

The main methods for producing metallic NP are based on chemical and physical approaches, which are often costly and generate toxic by-products that pose risks to the environment (Toranzo et al., 2021). Consequently, increasing attention has been directed toward alternative approaches collectively known as “Biological or Green Synthesis” (Ledezma et al., 2014; Toranzo et al., 2021). These methods use bacteria, fungi or plant extracts as natural reducing agents of the metal involved (Asif Asghar et al., 2018). Plant extracts are particularly attractive because they contain a wide variety of biomolecules, including phenolic compounds (e.g., flavonoids, hydroxycinnamic acids, and tannins), alkaloids, terpenoids, and polysaccharides, which can reduce metal ions to metallic NPs while simultaneously acting as stabilizing agents. As a result, green synthesis is typically a rapid, one-step process that can be carried out under ambient temperature and pressure, making it environmentally friendly, cost-effective, and readily scalable (Asif Asghar et al., 2018; Quinteros et al., 2019). Although several plant species have been reported for the green synthesis of metallic NP, much remains to be explored (Amooaghaie et al., 2015).

Despite the growing interest in ultrasound-assisted extraction as a sustainable technology for the recovery of bioactive compounds, its application to *F. bidentis* has received little attention. To the best of our knowledge, no studies have reported the optimization of ultrasound-assisted aqueous extraction of phenolic compounds from *F. bidentis* biomass, or the direct use of the resulting extracts as reducing and stabilizing agents for the green synthesis of silver nanoparticles. Consequently, the potential of this species as a source of phenolic compounds for integrated extraction–nanoparticle production processes remain largely unexplored. To address this knowledge gap, this study proposes ultrasound-assisted aqueous extraction as a sustainable strategy for the recovery of phenolic compounds from *F. bidentis* biomass, followed by the direct use of the resulting extracts in the green synthesis of silver nanoparticles within an integrated process. The effects of extraction time (10–70 min), temperature (20 °C–80 °C), and solvent-to-sample ratio (10–20 mL/g) on the phenolic content (PC) of the extracts were investigated, and the extraction conditions were optimized using Response Surface Methodology (RSM) based on a three-factor Doehlert experimental design. Experimental validation confirmed that the optimized extraction conditions were reproducible and provided significantly higher phenolic recoveries than conventional extraction methods. The aqueous extracts obtained under the optimized extraction conditions were directly employed as reducing and stabilizing agents for silver nanoparticle synthesis, yielding predominantly spherical nanoparticles with nanometric dimensions and marked antibacterial activity against both Gram-positive and Gram-negative bacteria.

The main contribution of this work lies in coupling the extraction of phenolic compounds (phenolic acids and flavonoids, mainly glycosylated and sulfated–the latter being rare in nature–) with the subsequent green synthesis of silver nanoparticles using the phenolic-rich extracts, without intermediate purification steps. This integrated approach simplifies the overall process and demonstrates that the recovered phytochemicals can simultaneously act as bioactive compounds and as reducing and stabilizing agents for nanoparticle synthesis, providing an efficient and sustainable route to produce antimicrobial nanomaterials.

## 2 Materials and methods

### 2.1 Vegetal material

Aerial parts of *Flaveria bidentis* were collected during the flowering period along the Cruz del Eje–Deán Funes route, province of Córdoba, Argentina, between February and March 2024. Its botanical identification was carried out by the Instituto Multidisciplinario de Biologia Vegetal-IMBIV-CONICET (voucher model number 2813 deposited in CORD). Leaves and flowers were dried at 100 °C–105 °C in a drying oven (SJ, model SL30, Argentina) until reaching a moisture content of 4.9%–5.6%, as determined by the gravimetric method. This drying was applied to ensure rapid moisture removal and biomass standardization prior to extraction. Nevertheless, drying at 100 °C–105 °C may have reduced the content of some thermolabile phenolic compounds, and the phenolic profile obtained therefore corresponds to the fraction remaining after the drying process. Both aerial parts were mechanically ground (Retsch knife mill KG 5657 HAAN, West Germany) and sieved through a standard No. 16 mesh sieve to obtain particles ranging in size from 420 to <1,190 μm.

### 2.2 Chemicals and reagents

Distilled water was used as the extraction solvent. The gallic acid (GA) standard was obtained from Sigma-Aldrich, sodium carbonate from J.T.Baker, and the Folin-Ciocalteu reagent from Fisher Chemical. AgNO_3_ from Sigma-Aldrich was used for the synthesis of the NP.

### 2.3 Ultrasound-assisted extraction

**FIGURE 1:**
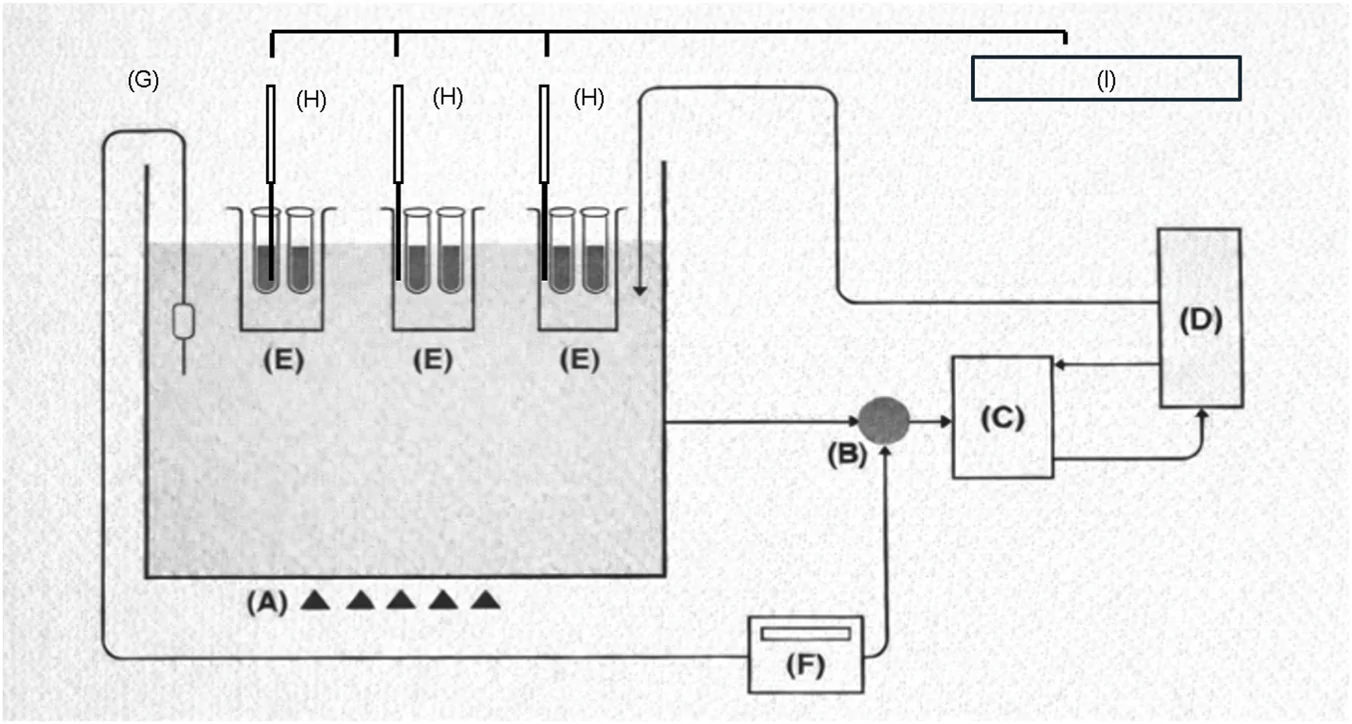
Experimental setup for power ultrasound assisted extraction experiments of phenols from *F. bidentis.* **(A)**, ultrasonic bath; **(B)**, pump; **(C)**, heat exchanger; **(D)**, cooler, **(E)**, sample tube holder; **F**, temperature controller; **(G)**, temperature sensor; **(H)**, ultrasonic transducer; **(I)**, ultrasonic generator.

All extractions were carried out in a steel ultrasonic bath (Clangsonic TU0740, China) with a maximum volume of 2.5 L (Figure 1). This equipment consists of three piezoelectric ultrasound transducers with a nominal power of 120 W and a nominal frequency of 39 kHz, a temperature controller with two external heaters, and a cooling system with a coil and flow pump. The control system keeps the temperature variation limited to ±1 °C during all experiments.

The plant material (0.5 g) was placed in a 50 mL transparent glass bottle containing distilled water according to the selected solvent/sample ratio (mL/g) (Table 1). The container was then immersed in a piezoelectric transducer coupled to a digital ultrasonic generator, maintaining the temperature (°C) and time (min) according to the conditions specified for each experiment (Table 1). The power applied per unit volume was 275 W/L. After the extractions, the plant material was separated from the liquid fraction by filtration, and the extracts were stored until further analysis.

**TABLE 1:** Doehlert’s experimental design together with experimental values.

Order of execution	A		B		C		Y	
	Time (minutes)		Temperature (°C)		Solvent/sample ratio (mL/g)		Yield of PC (mg GAE/g leaves)	Yield of PC (mg GAE/g flowers)
	Code	Level	Code	Level	Code	Level		
1	0	40	0	50	0	15	187.93	97.66
2	1	70	0	50	0	15	195.96	105.39
3	0.5	55	0.866	80	0	15	203.80	108.69
4	-0.5	25	0.866	80	0	15	199.66	108.43
5	-1	10	0	50	0	15	182.82	85.53
6	-0.5	25	-0.866	20	0	15	175.95	76.46
7	0.5	55	-0.866	20	0	15	182.96	83.65
8	0.5	55	0.289	60	0.816	20	244.91	134.52
9	-0.5	25	0.289	60	0.816	20	232.07	117.96
10	0	40	-0.577	30	0.816	20	209.84	112.03
11	0.5	55	-0.289	40	-0.816	10	134.20	68.98
12	-0.5	25	-0.289	40	-0.816	10	132.36	61.39
13	0	40	0.577	70	-0.816	10	171.95	88.00
14	0	40	0	50	0	15	184.70	99.41
15	0	40	0	50	0	15	185.89	100.53
16	0	40	0	50	0	15	184.64	98.20

### 2.4 Experimental design

An exhaustive literature search was carried out to determine the most influential factors in the UAE yields of PC from different plant matrices (Chen et al., 2017; Luo et al., 2018; Li et al., 2019; Setyaningsih et al., 2019; Oliveira Chaves et al., 2020; Biswas et al., 2023; Mihaylova et al., 2024; Savaş, 2024; Expósito-Almellón et al., 2025). Based on the literature analysis, three variables were selected for the UAE of PC from *F. bidentis* leaves and flowers: time (A, min), temperature (B, °C), and solvent/sample ratio (C, mL/g). To determine the optimal conditions within the investigated experimental domain and evaluate the effect of these variables, a Doehlert experimental design (Doehlert, 1970) was used with different levels for each of the chosen variables. Five levels were established for time (A), from 10 to 70 min; seven levels for temperature (B), from 20 °C to 80 °C; and three levels for solvent/sample ratio (C), from 10 to 20 mL/g. The investigated experimental ranges were deliberately selected considering both process and equipment feasibility and sustainability. In particular, because water was used as the sole extraction solvent, the upper temperature limit was established below its boiling point to minimize solvent evaporation, maintain stable ultrasonic cavitation conditions, and reduce the risk of thermal degradation of phenolic compounds. The investigated extraction time range was selected considering energy consumption, whereas the solvent-to-solid ratio range was defined based on literature reports while minimizing excessive solvent consumption and the associated downstream handling and processing requirements. Thus, the experimental matrix included sixteen tests, three correspond to replicates at the center point of the experimental domain (Table 1). The PC extraction yield was chosen as the dependent variable (Y) or response in the experimental design. The yield was related to the independent variables (A, B, C) by the following empirical quadratic polynomial model (Equation 1):

$$Y=b_{0}+b_{1}A+b_{2}B+b_{3}C+b_{11}A^{2}+b_{22}B^{2}+b_{33}C^{2}+b_{12}\text{AB}+b_{23}\text{BC}+b_{13}\text{AC}$$ (1)

where b_0_ is a compensation term, b_1_, b_2_, b_3_ are linear effects, b_11_, b_22_, b_33_ are quadratic effects and b_12_, b_23_, b_13_ are interaction terms.

Statgraphics Centurion XVI software (v16.1, USA) was used for data analysis. Model adequacy and statistical significance of regression coefficients were tested using ANOVA, considering them significant with a p-value <0.05 and a F-value calculated higher than the tabulated F-value (critical value: 4.10) (Montgomery, 2013). The coefficient of determination *R* ^2^ and the *R* ^2^ adjusted (R^2^_adj_) were used to evaluate the goodness-of-fit of the polynomial model equation. Response surface curves were used to assess interactions between independent variables and their effect on the response. Optimal conditions were determined by maximizing the desirability function.

To validate the extraction conditions provided by the predictive model, determined using the desirability function, we conducted a validation experiment using adjusted extraction conditions (Table 2). These adjustments were based on the on the operating characteristics of the equipment to ensure practical implementation. Three experiments (n = 3) were performed under these conditions. Agreement between theoretical and experimental values was confirmed when the residual (deviation between the two values) fell within the model’s error margins (Mean Squared Error - RMSE) (Bezerra et al., 2008).

**TABLE 2:** Optimal conditions, theoretical and experimental yields of the PC extraction process.

Parts of the plant	Optimal experimental conditions			Theoretical	Experimental
	Time (min)	Temperature (°C)	Solvent sample ratio (mL/g)	Yield of PC (mg GAE/g vegetal material)	Yield of PC (mg GAE/g vegetal material)
Leaves	68	59	20	245.19	250.86 ± 1.13
Flowers	65	75	20	136.18	137.37 ± 1.21

### 2.5 Phenolic compounds content determination

The PC content of the extracts was determined using the Folin-Ciocalteu technique, with some modifications (Xavier et al., 2024). Although the Folin-Ciocalteu reagent is not specific for phenolic compounds and may also respond to other reducing substances present in the sample, the assay was originally developed by Singleton and Rossi (1965) for the determination of total phenols and remains widely used as an estimate of total phenolic content, expressed as gallic acid equivalents (GAE), despite its recognized analytical limitations (Singleton and Rossi, 1965). Briefly, the extracts were diluted (1/30 for leaves and 1/15 for flowers). Then, to an aliquot of each dilution (130 μL) 650 μL of the previously diluted Folin-Ciocalteu reagent (1/10 in water) and 520 μL of a Na_2_CO_3_ solution (7.5% W/V) were added to an aliquot of each dilution (130 μL). The samples were placed in a thermostatic water bath at 50 °C for 5 min and subsequently incubated in the dark for 15 min at room temperature. Finally, their absorbance was measured at 760 nm using a Shimadzu UVmini-1240 spectrophotometer.

PC was quantified in each sample using an external standard calibration method. For this purpose, GA solutions ranging from 8.14 to 407.00 mg GA/L in distilled water were prepared, and their absorbance was measured in triplicate. The calibration curve was constructed by plotting the absorbance as a function of the GA concentration and the corresponding linear regression equation was obtained (correlation coefficients = 0.99). Accordingly, the PC yield was calculated for each sample using the following equation: Y = 0.011X, where Y represents the absorbance and X the GA concentration (mg GA/L). The PC yield results were expressed as GA equivalents (GAE) in mg per g of plant material dried extract and are shown in Table 1.

### 2.6 Synthesis and characterization of AgNP

AgNP were obtained by mixing an aqueous solution of silver nitrate (10 mM) with plant extract of *F. bidentis* leaves or flowers obtained under optimal point conditions of the experimental design (65/35% V/V ratio). The mixture was incubated in darkness for 15 min at room temperature. The formation of AgNP was confirmed by the color change and by UV–visible spectroscopy using a double-beam spectrophotometer (Thermo Electron Corporation - Evolution 300 BB UV-Vis Spectrophotometer, England) in the wavelength range of 200–600 nm at room temperature. The maximum absorbance peak observed (∼400 nm) is the characteristic surface plasmon resonance band of AgNPs. This optical phenomenon occurs during the bio-reduction of silver ions (Ag+) into colloidal nanoparticles (Toranzo et al., 2021). Subsequently, AgNP were characterized by transmission electron microscopy (TEM). To obtain TEM images, a 10 μL drop of nanosuspension was placed onto a formvar coated nickel grids and allowed to air dry. Finally, the specimens were examined using a Hitachi HT 7800 electron microscope (Hitachi, Tokyo, Japan) operated at 100 kV and photographed with a NS 15 AMT camera (Advanced Microscopy Techniques Corp., Woburn, MA, USA). The average size of the nanoparticles was determined from transmission electron microscopy (TEM) images. One hundred and fifty (150) particles per sample were analyzed using ImageJ software, with the image scale set using the scale bar provided in the micrograph. The data was exported to a spreadsheet to determine the average diameter and construct size distribution histograms. In addition, the ζ-potential of the AgNPs was determined by Dynamic Light Scattering (DLS) using a Malvern Instrument NANO ZS 90 equipment, and the results were analyzed with Zetasizer software version 7.12.

### 2.7 *In vitro* antibacterial activity

The antibacterial activity of the synthesized AgNP was evaluated *in vitro* by diffusion assay against three human pathogenic strains: *Staphylococcus aureus* ATCC 29213, *Pseudomonas aeruginosa* ATCC 27853*,* and *Escherichia coli* ATCC 25922. The diffusion assay was chosen because it is a robust qualitative indicator that not only confirms bioactivity but also the ability of nanoparticles to effectively diffuse into a solid matrix. Furthermore, the use of zones of inhibition as the primary metric is an accepted standard in green nanotechnology and has been reported in numerous high-impact studies to validate the efficacy of biogenic AgNPs (Chahar et al., 2018; Mohamed et al., 2024). For this, the microorganisms were reactivated on Müller-Hinton agar (MHA) and incubated for 24 h at 37 °C. A sample of each microorganism was then placed in sterile Mueller-Hinton broth and adjusted spectrophotometrically to the McFarland standard number 0.5 equals 1,5 × 10^8^ CFU/mL. Sterile disposable Petri dishes were prepared with 20 mL of MHA. Once the agar had solidified, the microorganisms were seeded by swabbing the solution uniformly over the agar surface. The positive control used in the assay was a gentamicin disk with a concentration of 10 μg, which was placed in the center of the agar plate. On the other hand, 40 µL of PBS solution and the same volume of leaf and flower extracts obtained under optimal conditions, used as a negative control, were placed in individual wells of Müller-Hinton agar. This procedure was also used for the corresponding synthesized silver nanoparticles (AgNPs). Then, after incubation at 37 °C for 24 h, in each case the inhibition zone was measured (diameter in mm), to evaluate the antibacterial activity (Onnainty et al., 2016). This experimental set was performed in triplicate.

## 3 Results

### 3.1 Optimization of UAE of PC

Table 1 shows the PC extraction yields under the 13 experimental conditions analyzed, together with the three replicates of the central point (total = 16 experiments). The regression model fit was assessed by *R* ^2^ (0.96 for leaves and 0.97 for flowers) and R^2^_adj_ (0.90 for leaves and 0.93 for flowers). These high coefficients indicate a strong agreement between the obtained and predicted responses.

Multiple regression models relating PC yield to the coded variables A (time), B (temperature), and C (solvent/sample ratio), including linear, interaction, and quadratic terms, are expressed by the following second-order polynomial equations (Equation 2: for the leaves; Equation 3: for the flowers):

$$Y=42.5796\;‐\;0.620702\;A+0.650004\;B+7.86471\;C+0.00399739\;A^{2}\;‐\;0.00158944\text{ AB}+0.0398276\text{ AC}+0.00433465\;B^{2}\;‐\;0.0307264\text{ BC}+0.0118398\;C^{2}$$ (2)

$$Y=-\;28.2258+0.11596\;A+1.04647\;B+6.04374\;C\;-\;0.00387875\;A^{2}+0.000501944\text{ AB}+0.0289134\text{ AC }-\;0.00201298\;B^{2}\;‐\;0.0269065\text{ BC }‐\;0.0327713\;C^{2}$$ (3)

An ANOVA test (F-value and p-value) was performed to evaluate model adequacy and fit. For the leaves, the model showed an F-value of 15.58 and a p-value of 0.0017, confirming statistical significance (see Supplementary Table 1). Similarly, for the flowers the model exhibited statistical significance, with an F-value = 24.21 and a p-value = 0.0005 (see Supplementary Table 2).

**FIGURE 2:**
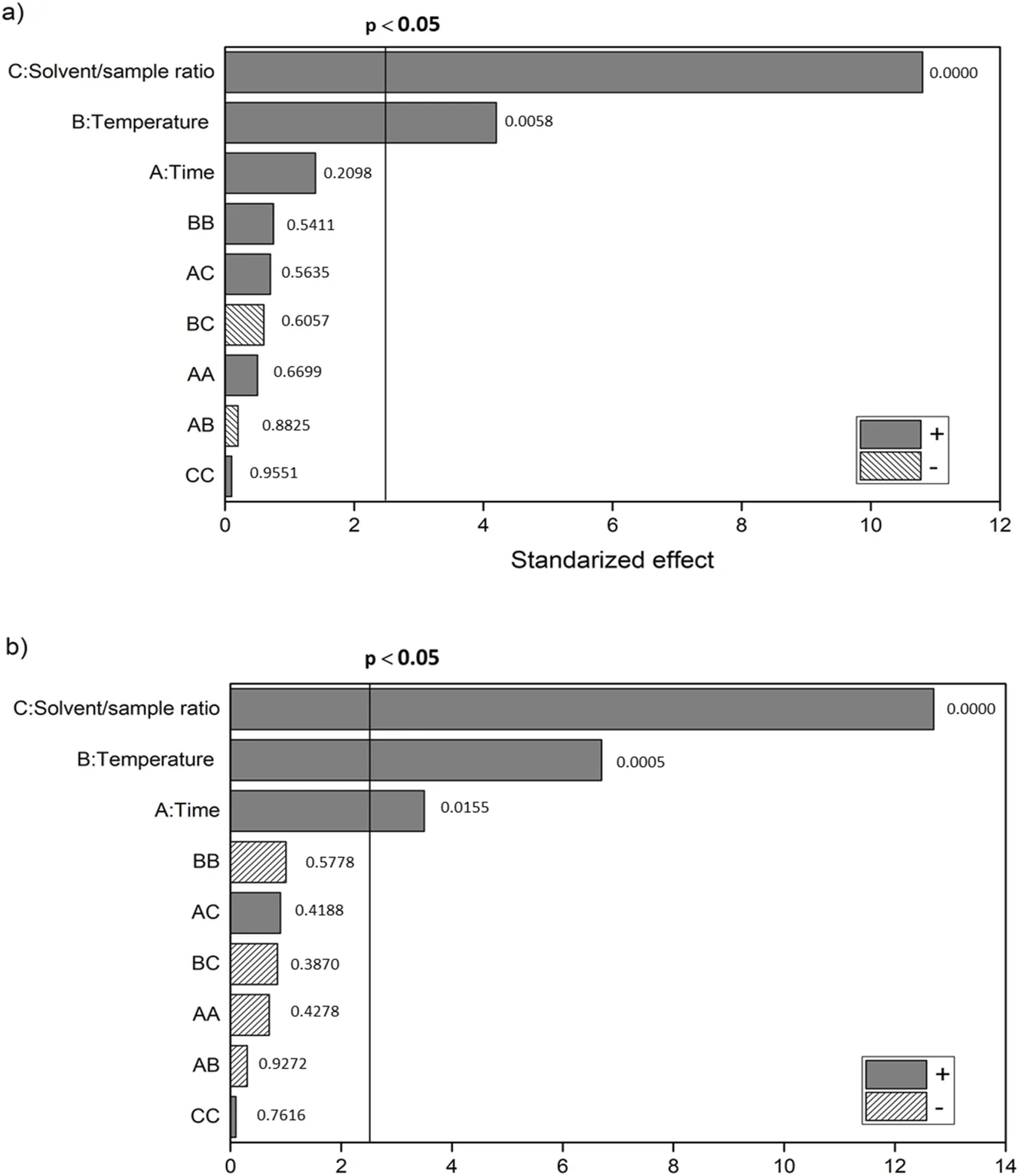
PC yield: Pareto chart of main effects obtained from Doehlert’s experimental design. **(a)** Leaves, **(b)** flowers. The vertical line defines the 95% confidence interval.

Also, the influence of each variable on PC yield was assessed by the ANOVA test (see Supplementary Tables 1, 2) and the results are presented in the Pareto charts (Figure 2). For leaves, the significant variables were B (temperature) and C (solvent/sample ratio) (p < 0.05) (Figure 2a). In flowers, A (time) was also significant (p < 0.05), in addition to temperature (B) and solvent/sample ratio (C), (Figure 2b); indicating that the independent variables were correctly selected.

In addition, this model also shows a positive relationship between the PC yield obtained by UAE and the linear terms of the significant variables (Figures 2a,b) in all cases. For both parts of the plant, the most influential variable in the PC extraction process was the solvent/sample ratio (C, the most influential factor–longest bar length in the Pareto chart).

**FIGURE 3:**
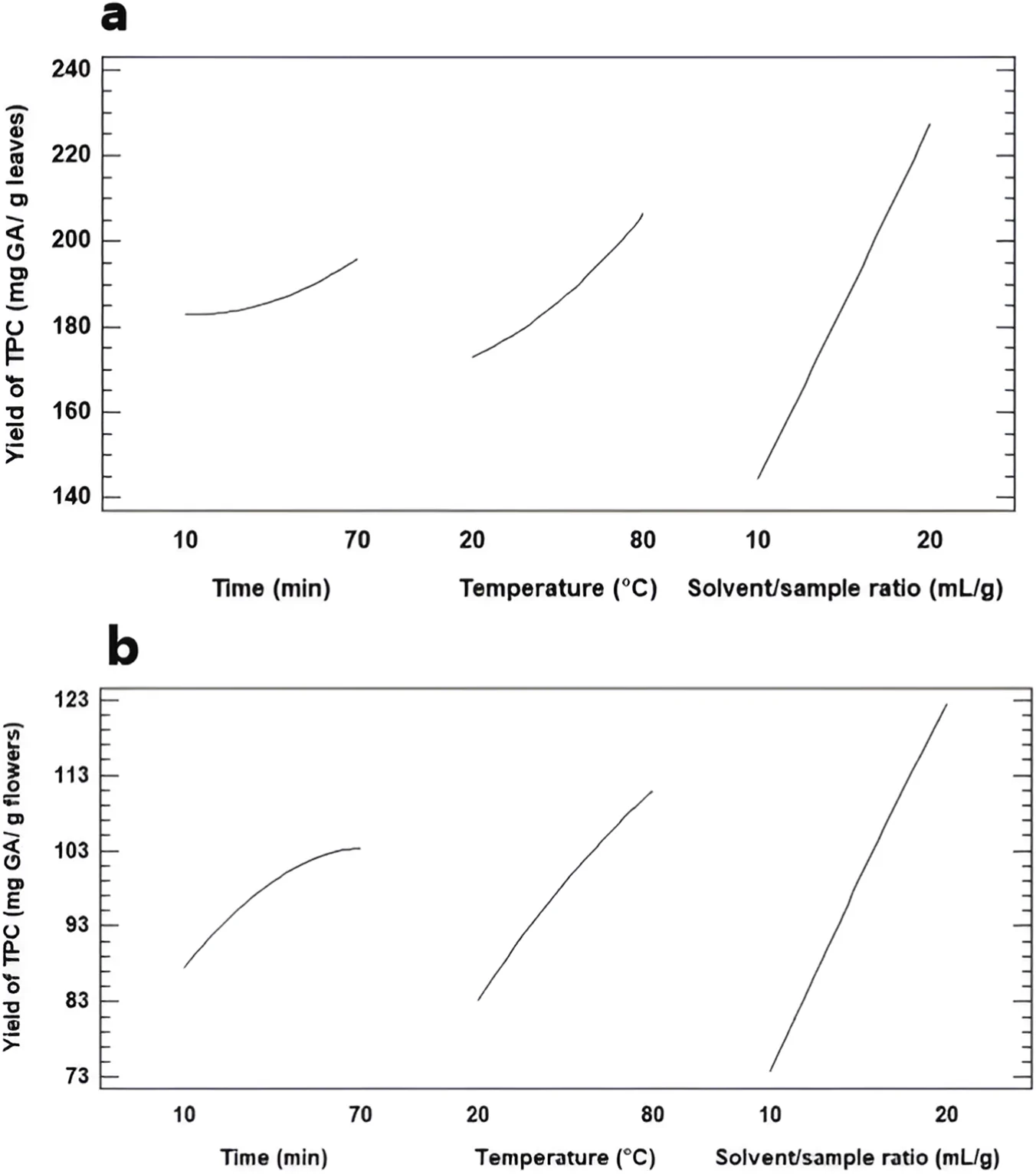
Main effects plots for PC extraction yields. **(a)** Leaves, **(b)** flowers.

**FIGURE 4:**
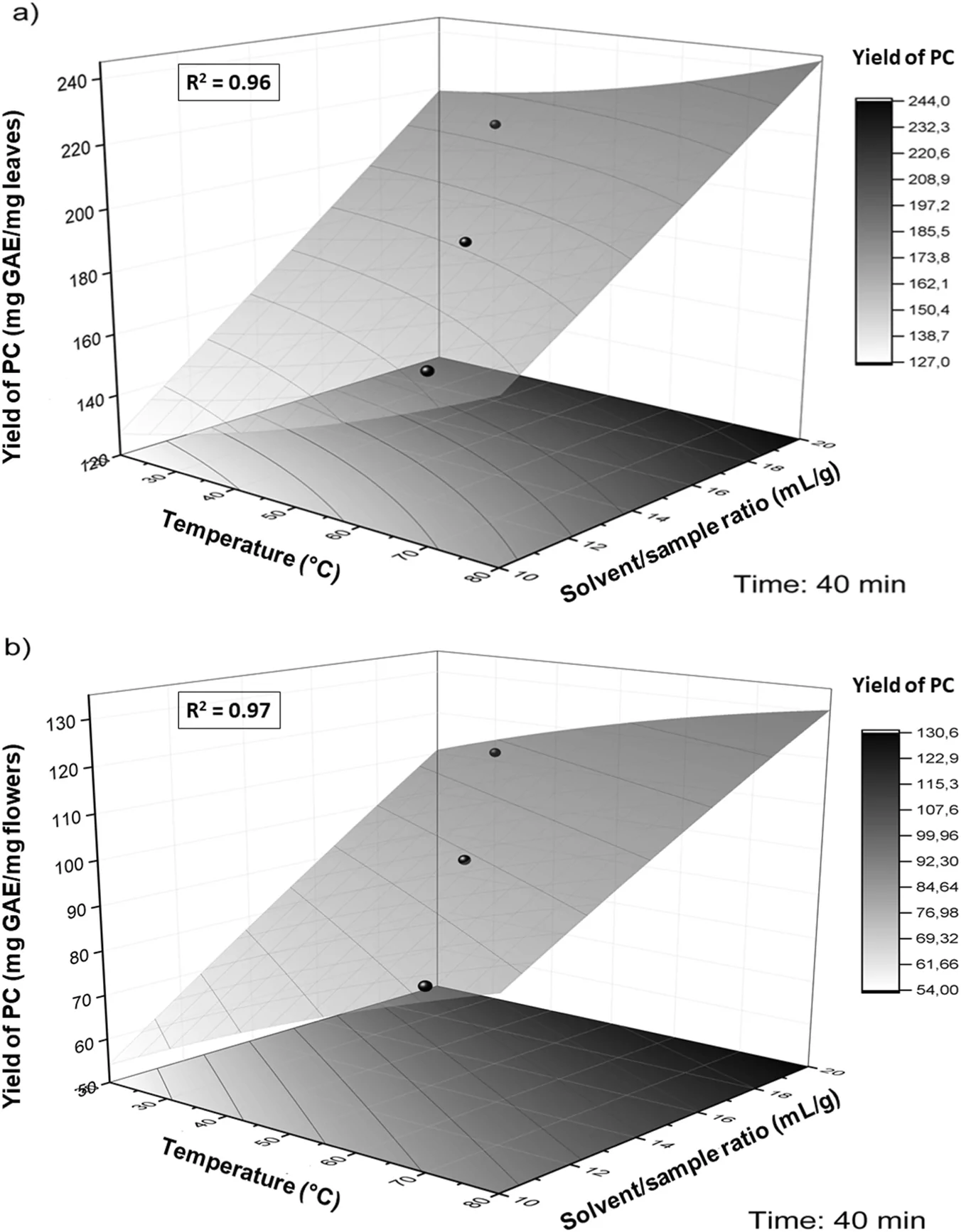
Three-dimensional response surface showing the combined effects of temperature and solvent/sample ratio at constant time (40 min) on PC yield: **(a)** leaves, **(b)** flowers. Experimental data points are represented by spheres.

Regarding the solvent/sample ratio (C), the positive coefficient of this term indicates that a higher solvent/sample ratio increases the PC extraction yield, for both parts of plants (Figures 3, 4). From a transport phenomena perspective, this behavior can be attributed to improved mass transfer conditions. At low solvent/sample ratios, the system tends to exhibit higher effective viscosity and limited solvent availability, restricting solvent infiltration into the plant matrix. In addition, early solvent saturation may occur, reducing the driving force for solute diffusion. Additionally, the higher viscosity could hinder the cavitation effect since the negative pressure during the rarefaction cycle must overcome a greater cohesive force (Kumar et al., 2021; El Baakili et al., 2023). As the solvent/sample ratio increases, the physicochemical environment becomes more favorable for extraction: the effective viscosity decreases, enhancing solvent diffusivity, while improved penetration into the porous plant structure promotes matrix swelling and facilitates internal mass transfer.

Moreover, the higher solvent volume reduces solute concentration in the bulk phase, maintaining a stronger concentration gradient that drives diffusion and reducing the saturation effect (Kumar et al., 2021). Altogether, these factors lead to enhanced solubilization and transport of phenolic compounds, resulting in higher extraction yields. Furthermore, the lower viscosity of extraction media produces a more intense cavitation effect (Kumar et al., 2021; El Baakili et al., 2023), contributing to greater disruption of the cellular structure and increased interfacial area between solvent and solute. Consequently, both external mass transfer and internal diffusion of phenolic compounds are improved Maran and Priya 2014; Xu et al., 2014; Samaram et al., 2015; He et al., 2016). Similar results were reported by Setyaningsih *et al.*, who observed an increase in the extraction yield of phenols from rice grains at the highest solvent/sample ratio tested (Setyaningsih et al., 2019). Other studies have also confirmed this observation for the extraction of phenols from different plant species (Chen et al., 2017; Jovanović et al., 2017; de Oliveira et al., 2019; Pinela et al., 2019).

A similar trend was observed for the linear term B (temperature), where the positive coefficient indicates that increasing temperature enhances PC extraction yield (Figures 3, 4). This effect can be explained by the combined influence of thermodynamic and transport phenomena during ultrasound-assisted extraction. On one hand, higher temperatures promote the disruption of plant matrix interactions, facilitating the desorption of phenolic compounds and increasing their solubility in the solvent (Medina-Torres et al., 2017). Moreover, increasing temperature enhances the solubility and diffusion coefficients of phenolic compounds, improving extraction (Cacace and Mazza, 2003). In addition, temperature reduces solvent viscosity and surface tension, which enhances solvent diffusivity and improves its penetration into the plant matrix (Celli et al., 2015; Wen et al., 2018; Kumar et al., 2021).

From a mechanistic standpoint, these changes also affect cavitation dynamics: lower surface tension and viscosity facilitate bubble formation and collapse, intensifying microjetting and localized shear forces that contribute to cell wall disruption (Chemat et al., 2017). As a result, both external mass transfer and internal diffusion of solutes are enhanced. Furthermore, the effect of temperature appears to be more pronounced in flowers than in leaves, likely due to differences in tissue structure and composition, which may lead to greater susceptibility to cavitation-induced disruption and improved solvent accessibility in the former. Altogether, these factors contribute to the observed increase in extraction efficiency with temperature.

**FIGURE 5:**
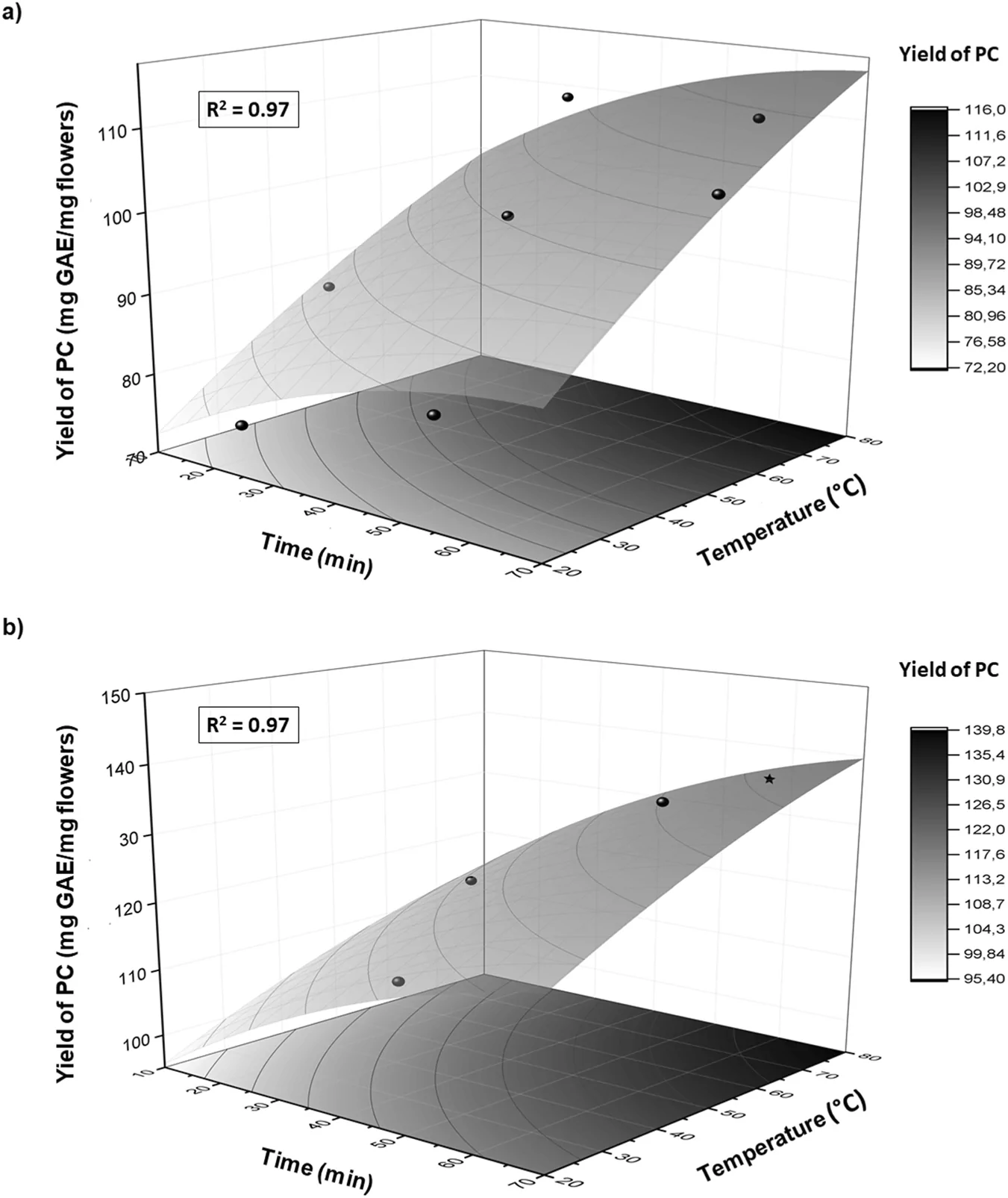
Three-dimensional response surface showing the combined effects of extraction time and temperature on PC yield from flower extracts at constant solvent/sample ratio: **(a)** 15 mL/g; **(b)** 20 mL/g. Experimental data points are represented by spheres, while the predicted optimal conditions are indicated by a star. The smooth surface corresponds to the fitted RSM model (*R* ^2^ = 0.97).

Particularly for flowers, the linear and significant term A: Time, was also found to be positive, indicating an directly proportional effect between the time and the extraction yield of the compounds of interest, through the use of UAE (Figures 2b, 3b, 5a,b). This could be because increasing the ultrasound time maintains a longer ultracavitation effect, thus improving hydration, fragmentation, and pore formation in the plant matrix, increasing the exposure of the solute and favoring its release into the extraction medium. This effect has also been reported for the extraction of phenolic compounds of grape pomace (González-Centeno et al., 2015), the phenolic compounds and anthocyanin of *Jabuticaba peel* (Rodrigues et al., 2015), among others.

Using the desirability function of response surface analysis, the optimal conditions to maximize PC extraction yield were determined (Table 2). For leaves, the optimal solvent/sample ratio was 20 mL/g, with an extraction time of 68 min and a temperature of 59 °C, resulting in a theoretical PC yield of 245.19 mg GAE/g of plant material. For flowers, the optimal solvent/sample ratio was the same (20 mL/g), with a similar time (65 min) and a temperature of 75 °C, obtaining a theoretical PC yield of 136.18 mg GAE/g of plant material (Figure 5b). The variations in phenolic content between leaves and flowers could be due to the influence of the different botanical and chemical properties inherent in each plant organ (Zagoskina et al., 2023).

For both plant parts, the optimal values for solvent/sample ratio and extraction time are high and similar. This behavior suggests that phenolic compounds extraction depends on these operating conditions, which are indeed significant, rather than on the specific plant part. However, the optimal extraction yield for flowers was obtained at a higher temperature than that required for leaves. This difference can be attributed to variations in the structural and chemical composition between the two parts of the plant. For temperature and extraction time, the predicted optimum was located within the investigated experimental domain rather than at its upper boundaries. Although temperatures up to 80 °C and the maximum extraction times defined in the experimental design were evaluated, the response surface models predicted optimal conditions of 59 °C and 68 min for leaves and 75 °C and 65 min for flowers. These results indicate that the optimum resulted from the combined effects and interactions among the extraction variables rather than from simply maximizing each factor independently.

In contrast, the optimum solvent-to-solid ratio corresponded to the highest value investigated (20 mL g^-1^), suggesting that higher ratios could potentially further increase PC recovery. Nevertheless, the selected range was intentionally limited because increasing the solvent-to-solid ratio would inevitably increase water consumption, reduce process efficiency, and increase the volume of extract requiring downstream handling or processing, which would be contrary to the objective of developing a simple, sustainable, and readily scalable extraction process using water as the sole solvent. Therefore, the reported solvent-to-solid ratio should be interpreted as the optimum within the investigated and technologically relevant experimental domain. Nevertheless, the influence of higher solvent-to-solid ratios deserves further investigation and will be addressed in future studies.

Finally, to validate the reliability of the predicted optimal extraction conditions, three extraction experiments were performed under the optimal conditions (Table 2), and the experimental values were 250.86 ± 1.13 mg/g and 137.37 ± 1.21 mg/g for leaves and flowers, respectively. The absolute deviations from the theoretical optimum were 5.67 and 1.19 for leaves and flowers, respectively. Both values were lower than the RMSE (9.27 and 4.74 for leaves and flowers, respectively), confirming the good predictive ability of the model. These results confirm the predictive capacity of the model and its suitability for determining the optimal extraction conditions within the experimental domain, despite the lack of significant fit (p = 0.0027 and 0.0371 for leaves and flowers, respectively) (Khumpirapang et al., 2020) (see Supplementary Tables 1, 2).

### 3.2 AgNP biosynthesis and characterization

**FIGURE 6:**
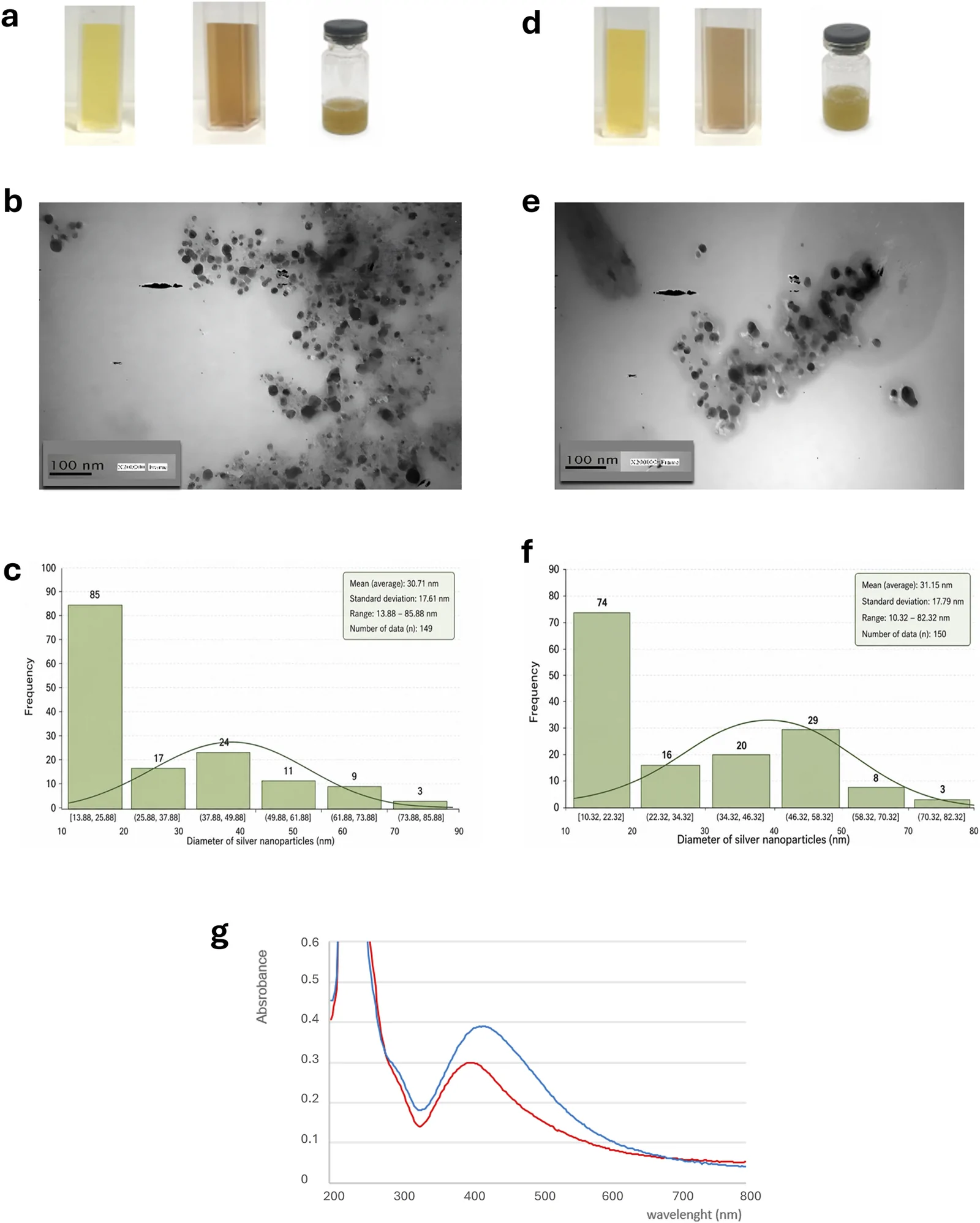
Characterization of silver nanoparticles (AgNPs) synthesized using leaf and flower extracts. **(a)** Photographs of the leaf extract before and after AgNP synthesis and the nanoparticle suspension. **(b)** Representative transmission electron microscopy (TEM) image of leaf-mediated AgNPs. **(c)** Particle size distribution histogram of leaf-mediated AgNPs. **(d)** Photographs of the flower extract before and after AgNP synthesis and the nanoparticle suspension. **(e)** Representative TEM image of flower-mediated AgNPs. **(f)** Particle size distribution histogram of flower-mediated AgNPs. **(g)** UV–Vis absorption spectra of AgNPs synthesized using leaf (blue line) and flower (red line) extracts, showing the characteristic surface plasmon resonance band.

The phenolic compounds-enriched extracts obtained during optimized UAE were used directly, without further purification, for AgNP synthesis. During this process, the phenolic compounds are oxidized, allowing the reduction of the Ag^+^ present in solution and, consequently, resulting a gradual color change from light yellow to dark brown (Figures 6a,d). This may be due to the specific optical property of surface plasmon resonance, which indicates the formation of AgNP (Suresh et al., 2014). Various studies on AgNPs biosynthesis using plant extracts, such as banana peel (Ohiduzzaman et al., 2024), lemongrass or *Cymbopogon citratus* (Rakib-Uz-Zaman et al., 2022), *Cannabis sativa* leaves (Csakvari et al., 2021) and cilantro (Atrooz et al., 2023), have reported similar behavior. Furthermore, Figure 6g shows that the UV-visible absorption spectra exhibited an absorbance peak around 400 nm, indicating the bioreduction of Ag^+^ ions from AgNO_3_ to AgNPs. The AgNPs synthesized from leaf extracts display a more intense and broader SPR band centered at approximately 420 nm, whereas those synthesized from flower extracts show a lower-intensity band at the same wavelength, suggesting differences in nanoparticle concentration and/or size distribution between the two biosynthetic routes.

The same figure shows the TEM images and histograms of the AgNPs synthesized using extracts from leaves (Figures 6b,c) and flowers (Figures 6e,f). In both cases, the AgNPs displayed a predominantly spherical morphology, with an average particle size of 30.71 ± 17.61 and 31.15 ± 17.79 nm for AgNPs synthesized from the leaf and flower extracts, respectively. Furthermore, DLS analysis showed that the ζ-potential of these AgNPs was negative (−15.0 mV and −12.1 mV for AgNPs synthesized from the leaf and flower extracts, respectively) (see Supplementary Figure 1), which suggest a modest colloidal stability (Pungle et al., 2022).

### 3.3 Antimicrobial activity

Table 3 and Supplementary Figure 2 shows the data obtained from the agar diffusion test. In Table 3 the results are expressed as zones of inhibition produced by the synthesized AgNPs against *S. aureus, P. aeruginosa*, and *E. coli*. The results demonstrate that AgNPs synthesized using plant extracts from leaves and flowers exhibited robust and broad-spectrum antibacterial activity against the Gram-positive (*S. aureus*) and Gram-negative (*P. aeruginosa* and *E. coli*) strains evaluated, confirming that the high density of phenolic compounds extracted from *F. bidentis* using optimized UAE enables the production of functional nanomaterials. The optimized aqueous extracts were used without further purification as reducing and stabilizing agents in a one-step green synthesis process, and the resulting nanosuspensions were analyzed without centrifugation or washing. Although the potential contribution of residual phytochemicals was considered, the leaf and flower extracts rich in phenolic compounds, analyzed separately as negative controls, showed no inhibition zones, confirming that the phytochemicals alone were not responsible for the antibacterial effect at the concentrations used. Nevertheless, the presence of these phytochemicals could act as a coating agent, thereby becoming an integral and functional component of the final bioactive material. In these processes, polar metabolites, such as phenolic compounds, act simultaneously as reducing and natural coating agents. This dual function is crucial, as the phytochemicals stabilize the nanoparticles, preventing aggregation and preserving or enhancing their bioactivity. Finally, the comparable antibacterial activity of the biosynthesized AgNPs and AgNO_3_ should not be interpreted as evidence of complete nanoparticle dissolution. Since the antimicrobial activity was evaluated using an agar well diffusion assay, the observed inhibition zones reflect the combined effects of nanoparticle diffusion through the agar matrix, interactions between AgNPs and bacterial cells, and the possible release of Ag^+^ ions. Consequently, the relative contribution of each mechanism cannot be determined from the present experimental design.

**TABLE 3:** Antibacterial activity of AgNP against strains of *S. aureus*, *P. aeruginosa* and *E. coli.*

Samples	*S. aureus*	*P. aeruginosa*	*E. coli*
	Zone of inhibition (mm ± SD)	Zone of inhibition (mm ± SD)	Zone of inhibition (mm ± SD)
Phosphate-buffered saline (Negative control)	Without inhibition	Without inhibition	Without inhibition
Leaf extract	Without inhibition	Without inhibition	Without inhibition
Flower extract	Without inhibition	Without inhibition	Without inhibition
Gentamicin (Positive control)	20.3 ± 6	24.7 ± 6	25.0 ± 0
AgNO_3_ (ac, 10 mM)	23.0 ± 0	23.3 ± 6	20.0 ± 1
AgNP obtained from leaf extract	23.3 ± 6	23.0 ± 0	20.3 ± 6
AgNP obtained from flower extract	23.7 ± 6	24.0 ± 1	21.7 ± 6

SD = Standard deviation. All experiments were performed in triplicates and reported as means ± SD.

## 4 Discussion

This study proposes an integrated strategy for the valorization of biomass from the invasive plant *F. bidentis*, combining sustainable extraction processes with the generation of value-added bioactive materials. A key finding was the high recovery of phenolic compounds using water as the sole extraction solvent. The use of response surface methodology involving the Doehlert experimental design enabled the identification of the operating conditions that maximized recovery of phenolic compounds while maintaining an environmentally friendly extraction approach. Beyond enhancing extraction efficiency, the optimization of this initial stage was essential to provide phenolic-rich extracts suitable for subsequent proccesing steps.

In this framework, and following an extensive review of the available literature, the phenolic compounds yield obtained from *F. bidentis*, particularly in the leaves (250.86 mg/g), can be considered noteworthy compared with values reported for UAE of botanical matrices. Although comparisons among studies should be interpreted with caution due to differences in plant materials, extraction conditions, solvents, analytical methodologies, and reporting units, the available literature provides a useful context for assessing the extraction performance achieved in the present work.

For example, green tea (*Camellia sinensis*), a well-established commercial source of phenolic compounds, has been reported to yield approximately 44.82 mg/g under UAE conditions (Khan et al., 2025). Likewise, higher or comparable values have been reported for coconut mesocarp (289.48 mg/g using 60% acetone; Yang et al., 2021), *Phyllanthi Fructus* (213.49 mg/g using 60% ethanol; Che et al., 2024), and *Retama sphaerocarpa* (154.09–160.15 mg/g using 58% ethanol; El Baakili et al., 2023). It is important to note that these studies employed organic or hydroalcoholic solvents, whereas in this work the phenolic compounds were obtained using distilled water as the sole extraction solvent. Furthermore, when compared with other optimized aqueous UAE processes, such as that reported for *Gnaphalium affine* (218.35 mg/g; Luangsakul et al., 2025), the values obtained for *F. bidentis* fall within the upper range of those reported in the literature.

Regarding organ differentiation, the phenolic compound yield obtained from flowers (137.37 mg/g) is also noteworthy among the values reported for floral matrices, exceeding that reported for *Clitoria ternatea* flowers (27.42 mg/g; Santos and Martins, 2023). It is also important to consider differences in the basis used to express the extraction yields. For instance, although Minhaj et al. (2025) reported 244.91 mg/g for *Opuntia ficus-indica* flowers, this value was expressed as milligrams per Gram of processed extract, whereas the results presented for *F. bidentis* are expressed as milligrams per Gram of dry plant material. Similarly, a polyphenol yield of 232.5 mg/g has been reported for *Polianthes tuberosa* (Yadav et al., 2023); however, this result was achieved using a combined ultrasound–microwave extraction process rather than UAE alone. Taken together, these observations indicate that *F. bidentis* is a promising source of phenolic compounds and that the extraction yields obtained in this work are among the highest values reported in comparable UAE-based studies, particularly considering that water was used as the only extraction solvent. The remarkable improvement in yield observed despite using distilled water as the sole solvent is especially significant, given that aqueous systems are typically associated with lower extraction efficiency. Our findings demonstrate that, under optimized ultrasound conditions, water can achieve superior extraction performance, eliminating the need for organic solvents. This 100% aqueous approach minimizes environmental impact and operational risks because of it is non-toxic and cost-effective (Chemat et al., 2017). Regarding the green synthesis of biogenic silver nanoparticles generated, it is important to note that the particle size (≈30 nm) is comparable with other biogenic nanoparticles; for example, it is larger than that AgNPs synthesized using tropical tea extracts such as *C. ternatea* (5.9 nm), Jasminum sambac (10.5 nm), and Hibiscus sabdariffa (16.1 nm) (Rizki et al., 2026). However, they are smaller than those AgNPs synthetized from extract of *Centella asiatica* (35–100 nm), the combined extract of *Petroselinum crispum* and *Piper betle* (126.3 nm) or *Persicaria hydropiper* (114.5 nm) (Arulnangai et al., 2025; Diab et al., 2025; Talukder et al., 2025). Furthermore, the AgNPs synthetized from *F. bidentis* extracts are predominantly spherical, exhibiting a uniform morphology, unlike other biogenic nanoparticles such as those from *C. asiatica*, which produced tubular and cuboidal shapes (Arulnangai et al., 2025).

In addition, it is important to highlight that these AgNPs were obtained through the direct use of phenolic-rich aqueous extracts by optimized ultrasound-assisted extraction from *F. bidentis*—without intermediate purification steps—as reducing and stabilizing agents in the green synthesis of these silver nanoparticles. In this system, water-extracted polar metabolites, such as phenolic compounds, facilitate the rapid and efficient reduction of silver ions, while also acting as natural coating agents (Amooaghaie et al., 2015). Moreover, *F. bidentis*-derived nanoparticles exhibited a consistent particle size across different plant organs (leaves vs. flowers), suggesting a robust synthesis process. Finally, when comparing the antimicrobial efficacy of the AgNP with the reference antibiotic, gentamicin (positive control), promising performance is observed. Specifically, AgNP obtained from the flower extract exhibited zones of inhibition that were comparable to those of gentamicin against *P. aeruginosa*. The comparable antibacterial activity observed in the diffusion assays indicates that these synthesized AgNPs possess promising antimicrobial properties. However, these findings should be considered preliminary, and further studies are required to confirm their potential applications. Finally, while both botanical sources produced functional AgNPs, the flower extracts generated AgNP with slightly greater antibacterial efficacy across all three tested strains (larger inhibition zones). This difference suggests variations in the reducing and stabilizing agents present in flowers compared to leaves, which could influence the biological activity of the nanoparticles produced.

Therefore, beyond the individual results, the main contribution of this work lies in its integrative and cascade nature: it connects sustainable extraction, the relevant recovery of high-value metabolites, and the production of functional nanomaterials in a single integrated, unified, and potentially scalable process, transforming waste of the *F. bidentis* biomass into high-value-added products.

## Data Availability

The original contributions presented in the study are included in the article/Supplementary Material, further inquiries can be directed to the corresponding author.
