# Supplementary material for: Sustainable and integrated cascade valorization of the biomass generated by the invasive plant Flaveria bidentis: optimization of ultrasound-assisted phenols extraction coupled to the green synthesis of biogenic antimicrobial silver nanoparticles
Source: Front Bioeng Biotechnol. 2026 Aug 19;14:1872480. doi: 10.3389/fbioe.2026.1872480 (PMC13534056; doi:10.3389/fbioe.2026.1872480)

SUPPLEMENTARY MATERIAL (SM)

Table I. ANOVA: Relationship between the independent variables and the response variable (UAE yield of PC of leaves).

Model term	Sum of squares	Degree of freedom (df)	Mean square	*F*-value	*p*-value
Time (A)	152.285	1	152.285	1.86	0.2216
Temperature (B)	1592.69	1	1592.69	19.45	0.0045 [a]
Solvent/sample ratio (C)	10275.5	1	10275.5	125.51	0.0000 [a]
AA	9.31393	1	9.31393	0.11	0.7474
AB	0.00458329	1	0.00458329	0.00	0.9943
AC	27.422	1	27.422	0.33	0.5838
BB	14.8599	1	14.8599	0.18	0.6849
BC	28.0654	1	28.0654	0.34	0.5795
CC	0.12058	1	0.12058	0.00	0.9706
Total error	491.209	6	81.8682		
Total (corr.)	12600.9	15			
Model	12061.99	9	1340.22	15.58	0.0017 [a]
lack-of-fit	508.83	3	169.61	71.66	0.0027[a]
**Statical Factors**					
R^2	0.96				
R^2_{adj}	0.90				
RMSE	9.27				

[a] significant at $p < 0.05$

Table II. ANOVA: Relationship between the independent variables and the response variable (UAE yield of PC of flowers).

**Model term**	**Sum of squares**	**Degree of freedom (df)**	**Mean square**	***F*-value**	***p*-value**
Time (A)	317.844	1	317.844	30.72	0.0015 [a]
Temperature (B)	1251.77	1	1251.77	120.99	0.0000 [a]
Solvent/sample ratio (C)	3560.19	1	3560.19	344.10	0.0000 [a]
AA	16.2483	1	16.2483	1.57	0.2568
AB	12.0378	1	12.0378	1.16	0.3222
AC	28.6704	1	28.6704	2.77	0.1470
BB	33.7315	1	33.7315	3.26	0.1210
BC	26.39	1	26.39	2.55	0.1614
CC	0.496585	1	0.496585	0.05	0.8338
Total error	62.0774	6	10.3462		
Total (corr.)	5296.22	15			
Model	4897.95	9	544.22	24.21	0.0005[a]
lack-of-fit	57.1297	3	19.0432	11.58	0.0371[a]
**Statical Factors**					
R^2	0.97				
R^2_{adj}	0.93				
RMSE	4.74				

[a] significant at $p < 0.05$

Fig. I. Zeta potential distribution of AgNP synthesized from leaf extract (a) and flower extract (b) of *F. bidentis*.

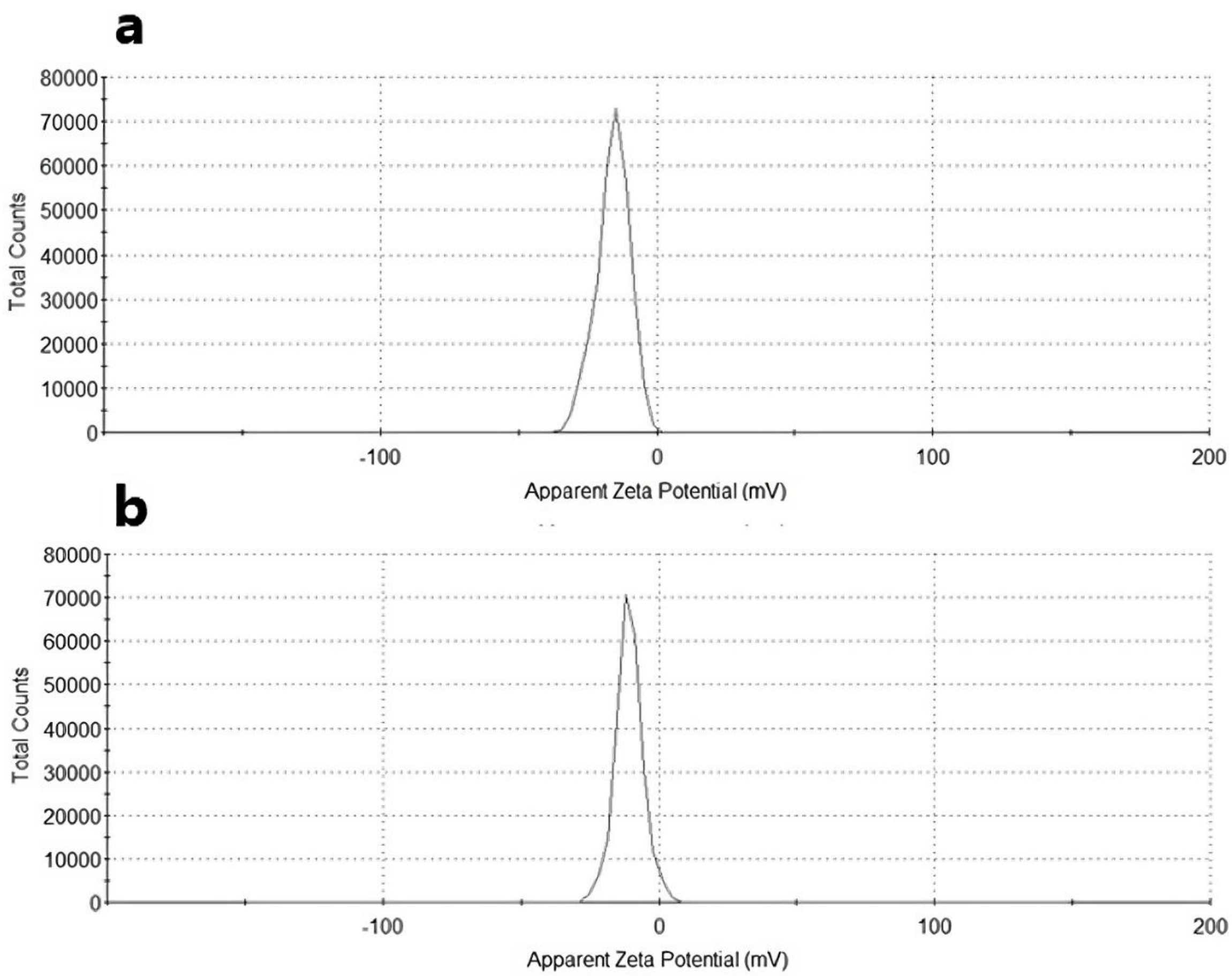

Fig. II. Representative images of the agar diffusion assays showing the antibacterial activity of silver nanoparticles (AgNPs). (a) *Pseudomonas aeruginosa*, and (b) *Escherichia coli*. Clear inhibition zones surrounding the wells indicate the antibacterial activity of the tested AgNP formulations, while differences in halo diameters reflect variations in antimicrobial effectiveness against each bacterial species. Images are representative of three independent experiments for each strain shown. The wells contained the different experimental treatments, including PBS (2), plant extracts (4, 5), biosynthesized AgNPs (6, 7), $AgNO_3$ solution (3), and the antibiotic positive control (1), as describes in Section 2.7.

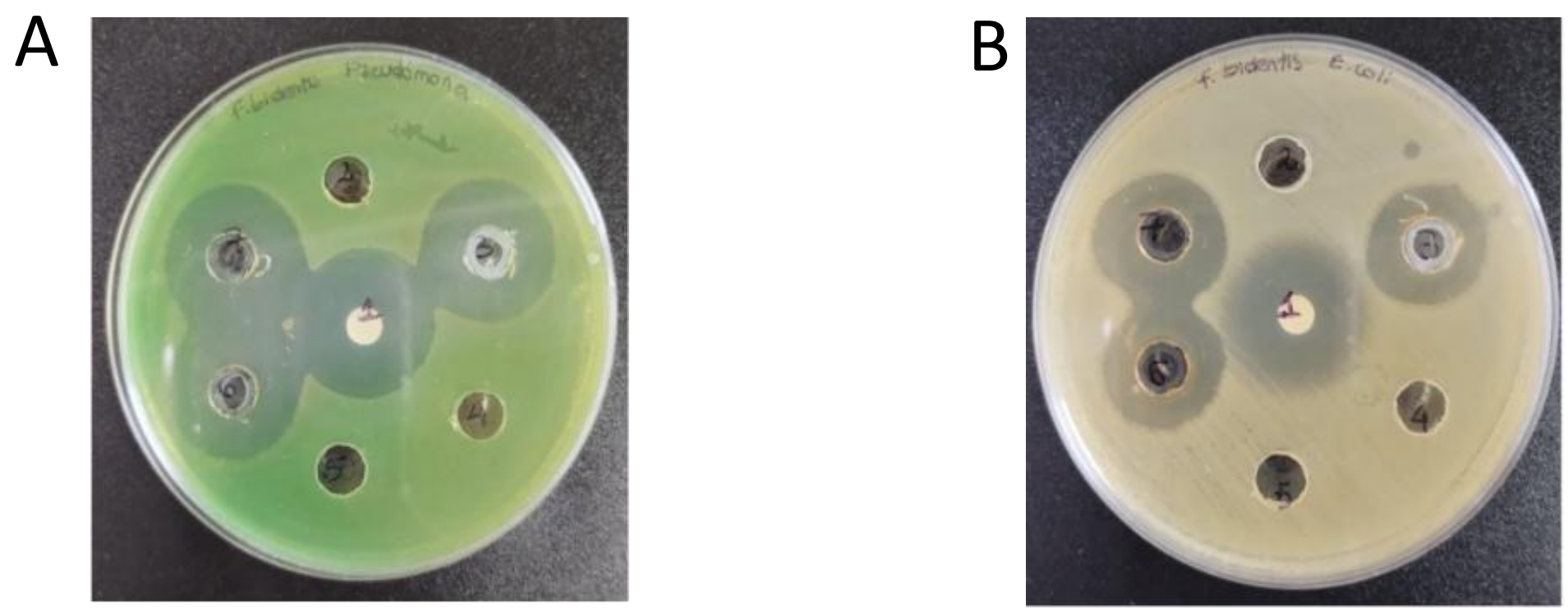

Supplement: Supplementary file 1 [file DataSheet1.pdf]
